# Routine Blood Examination Predicts the Course of Disease in Patients with Pseudoexfoliation

**DOI:** 10.3390/medicina61040652

**Published:** 2025-04-02

**Authors:** Tatjana Sarenac Vulovic, Katarina Cupic, Nenad Petrovic, Jovana Srejovic, Tatjana Vulovic, Zeljko Todorovic, Jovan Rakic, Dusan Todorovic

**Affiliations:** 1 Department of Ophthalmology, Faculty of Medical Sciences, University of Kragujevac, 34000 Kragujevac, Serbia; tvoja.tanja@yahoo.com (T.S.V.); nenadpet@yahoo.com (N.P.); srejovic.jovana@gmail.com (J.S.); drdusantodorovic@yahoo.com (D.T.); 2 Clinic of Ophthalmology, University Clinical Centre Kragujevac, 34000 Kragujevac, Serbia; 3 Department of Anesthesiology, Faculty of Medical Sciences, University of Kragujevac, 34000 Kragujevac, Serbia; tatjana_vulovic@yahoo.com; 4 Clinic of Anesthesiology, University Clinical Centre Kragujevac, 34000 Kragujevac, Serbia; 5 Department of Internal Medicine, Faculty of Medical Sciences, University of Kragujevac, 34000 Kragjevac, Serbia; todorovic_zeljko@hotmail.com; 6 Clinic of Hematology, University Clinical Centre Kragujevac, 34000 Kragujevac, Serbia; 7 Department of Dentistry, Faculty of Medical Sciences, University of Kragujevac, 34000 Kragujevac, Serbia; rakic999@hotmail.com

**Keywords:** pseudoexfoliation, glaucoma, blood cells, lipid profile, monocyte, platelet

## Abstract

*Background and Objectives*: Is it possible to predict the course of disease in patients with pseudoexfoliation based on blood examination? *Materials and Methods*: This retrospective study included 800 patients recruited for cataract surgery in the Clinic of Ophthalmology, University Clinical Centre Kragujevac, Serbia. The patients were divided into four groups: pseudoexfoliation syndrome early stage group (n = 200 patients), pseudoexfoliation syndrome late stage group (n = 200 patients), pseudoexfoliation glaucoma group (n = 200 patients) and the control group (n = 200 patients). During the preoperative process, some blood examination must be performed. We retrospectively used the results for the blood cell counts that we obtained from the patients. We recorded the neutrophil, lymphocyte, platelet, monocyte and leucocyte numbers, as well as the lipid profile, and simply calculated the ratio of their values, which we considered through different stages of the disease. *Results*: Our results indicated that there were no significant differences between all the groups examined in terms of leucocyte, neutrophil and lymphocyte count, but we recorded significant differences in the monocyte and platelet count. It was interesting that the monocyte count increased in the late stage of pseudoexfoliation syndrome and pseudoexfoliation glaucoma, in comparison with the control group and patients with early stage pseudoexfoliation syndrome. The lipid profile analysis indicated only increased values of LDL in patients with pseudoexfoliation (syndrome/glaucoma) in comparison with the control group. *Conclusions*: Monocytes are the main source of various cytokines, so our results support the proinflammatory theory of pseudoexfoliation development. Monocytes are the main cells in chronic inflammation, which leads to pseudoexfoliation syndrome. Platelets play an important role in the differentiation and activation of monocytes, as well as in the process of chronic inflammation and fibrosis, which are significant for pseudoexfoliation material production. A disturbed lipid profile in patients with pseudoexfoliation is expected, as they are at higher risk for cardiovascular and cerebrovascular diseases.

## 1. Introduction

Pseudoexfoliation syndrome (XFS) is a systemic age-related disorder [1]. Its presentation was demonstrated in different visceral organs and in the walls of the blood vessels [2]. In the eye it is described as a white granular deposition on the pupillary margin and on the lens anterior capsule. The origin of pseudoexfoliation (PEX) in the body is not yet clear. Different factors can induce the process of PEX production, such as oxidative stress, ultraviolet radiation, infection, etc. [2,3]. Based on its structure and immunohistochemical characteristics, it is supposed that PEX material is the final product of the general and local chronic inflammatory process [4,5,6].

Available data about the basic process of PEX production suggests that it is chronic inflammation with a default fibrotic process [7]. The main cell in chronic inflammation is the monocyte [8]. The proinflammatory and regulatory cytokine levels were disturbed in patients with XFS and pseudoexfoliation glaucoma (XFG), confirming this inflammatory theory. The determination of cytokine levels in the serum or other bodily fluids is very expensive and not feasible in everyday practice, so the use of routine blood examinations can be very valuable in this regard. Modern literature and practice suggest the use of certain inflammatory indices, which can be calculated very easily using the results of blood cell counts: NLR (neutrophil-to-lymphocyte ratio), PLR (platelet-to-lymphocyte ratio), LMR (lymphocyte-to-monocyte ratio), SII (systemic inflammatory index-neutrophil x platelet/lymphocyte count), SIRI (systemic inflammation response index-neutrophil x monocyte/lymphocyte count), etc. [9,10].

Routine blood examination always helps in different ways to predict different diseases. It can also be used for predicting XFS or XFG. Blood cell count is a very commonly performed test for almost every disease. The results give us so much information that can be used to predict inflammation, infection, tumors, ischemia, hypoxia, anemia, etc.

Dyslipidemia is known to be a very important risk factor for vascular disease. Patients with XFS/XFG can also have a disturbed lipid profile [11,12]. Lipid serum analysis can be used for prediction in patients with PEX.

The main aim of this clinical study was to evaluate the routine blood count as well as the lipid profile of patients with different stages of XFS and XFG, and of healthy subjects.

## 2. Materials and Methods

This retrospective study included 800 patients recruited for cataract surgery in the Clinic of Ophthalmology, University Clinical Centre Kragujevac, Serbia from 1 January 2020 to 31 December 2024. The patients were divided into four groups: I group—XFS early stage group (n = 200 patients), II group—XFS late stage group (n = 200 patients), III group—XFG group (n = 200 patients) consisting of the patients with XFG, and the control group (n = 200 patients). Participants were matched by sex and age. Excluded criteria were the diagnosis of ocular inflammation; the use of different ophthalmological drugs, except antiglaucomatous drugs without prostaglandin analogue; hematological disorders; cancer; systemic inflammatory diseases; chronic obstructive pulmonary disease; and the use of corticosteroid therapy in the past 3 months.

XFS and XFG were diagnosed using routine ophthalmological examination: visual acuity examination, intraocular pressure (IOP) measuring, detailed slit lamp examination in mydriasis, fundus biomicroscopy (90 D lens), gonioscopy and visual field examination. The presence of white flakes on the pupillary border and on the anterior capsule of the lens, as well as in the iridocorneal angle, was the deciding criteria for XFS. Early stage XFS includes the mild presence of PEX material on the anterior lens capsule and pupillary margin, maximally achieved 6 mm pupil dilation, initial pupillary atrophy and pigment dispersion in the iridocorneal angle [13]. Late stage XFS includes intensive PEX accumulation on the pupillary margin and anterior lens capsule, as well as pigmentation in the iridocorneal angle with present Sampaolesi line, maximally achieved 4 mm pupil dilatation and prominent iris atrophy. Elevated IOP (>21 mmHg), open iridocorneal angle with increased pigmentation, disturbed cup/disc ratio of the optic head with visual field defects, as well as the presence of PEX material at the pupillary margin and anterior lens capsule were diagnostic parameters for XFG.

During the preoperative process, some blood examination must be performed, including a complete blood cell examination. We retrospectively used the results for the blood cell counts that we obtained from the patients. We recorded the neutrophil, lymphocyte, platelet, monocyte and leucocyte numbers, as well as the lipid profile, and simply calculated the ratio of their values, which we considered through different stages of the disease. Venous blood was collected from an antecubital vein. Complete blood count (CBC) was performed within one hour after the blood collection. An automated blood cell counter (Mindray BC 3000 plus, Nanshan, Shenzhen, China) was used to analyze all cell types. The lipid levels in serum were measured using a Beckman Coluter 800/USA biochemical analyzer (Clinical Centre Kragujevac, Kragujevac, Serbia).

SPSS ver.22 was used for the statistical analysis of the results. The Kolmogorov–Smirnov test was used to check the normality of the distribution. If the distribution was not normal, the Kruskal–Wallis test was used for the analysis. Analysis of variance was used for the groups with normal distribution. Comparison between the groups was done using the Student’s *t*-test or the Mann–Whitney test for all continuous variables. Categorical variables were compared using the Chi-squared test. A value of *p* < 0.05 was considered to be statistically significant. Numerical results were presented as mean ± standard deviation.

## 3. Results

Our study included 800 patients, divided into four groups according to ophthalmological findings. The mean age of patients in I group was 68.1 ± 5.1, II group 65.6 ± 6.3, III group 67.1 ± 4.3, IV group 64.2 ± 5.2. Our patients were matched by age and sex.

Table 1 presents the mean count of different blood cells from the hemogram. We noticed that monocytes, lymphocytes and platelet count were statistically significantly disturbed in patients with PEX (syndrome/glaucoma).

Systemic immune indices were statistically significantly disturbed in PEX patients in comparison with the control group patients.

Our results indicated that there were no significant differences between all the examined groups in terms of leucocyte, neutrophil and lymphocyte count, but we recorded significant differences in monocyte and platelet count. Those differences were noted between all the PEX groups in comparison with the control group. It was interesting that the monocyte count increased in the late stage of XFS and XFG, with high statistical significance (*p* < 0.001) in comparison with control group, but with statistical significance in comparison to the patients with early stage XFS (*p* < 0.05), as well as the monocyte count of the patients with early stage XFS in comparison with the control group patients. Our results indicated a decreased trend of lymphocyte counts from early stage XFS or control groups in comparison with late stage of XFS and XFG, without significant differences.

Red cell distribution width (RDW) results indicated a significant increase in values in patients with late stage XFS and XFG in comparison with early stage XFS and the control group.

The lipid profile analysis indicated only increased values of LDL in PEX patients groups (XFS, XFG) in comparison with patients without PEX (*p* < 0.05; *p* < 0.001).

## 4. Discussion

Inflammation is the basic pathological process in numerous diseases, such as atherosclerosis, diabetes mellitus, cancer and Alzheimer dementia [14,15]. The great variety of tests can be performed to show evidence of chronic inflammation. Some of these tests are very expensive and very complicated to perform, so doctors make an effort to find an easy way to prove the existence of inflammation, such as a routine blood examination [16,17,18,19].

XFS is an age-related condition characterized by abnormal production and accumulation of PEX in the whole body, including in the eye [2]. Immunohistochemical analysis indicated that its structure was similar to fibrous tissue [4,5,6,20], and that the activity of some proinflammatory cytokines was increased in the process of XFS/XFG development [13,21]. XFS/XFG has some connection with different systemic manifestations: chronic heart decompensation, Alzheimer dementia, hearing loss and increased carotid resistance with low blood flow [2,11]. High sensitivity C reactive protein (CRP) was examined in the blood samples of the PEX patients with elevated results [22], but routine CRP examination did not indicate disturbed results [23].

Our results indicated statistically significant differences only in monocyte and platelet count in all PEX groups in comparison with the control group, especially in late stage XFS and in XFG. Monocytes are the main source of various cytokines, including IL-6 and IL-8 [24], the levels of which are higher in patients with PEX [7,21]. Our results indicated a decreased trend of lymphocyte counts from early stage XFS or in the control groups in comparison with late stage XFS and XFG, without statistically significant differences.

Bashir et al. recorded increased values of RDW in patients with PEX and with XFG, but as we divided patients with XFS into early and late stage, we found that patients with late stage had increased RDW values in comparison with patients with early stage XFS and the control group [25]. Increased RDW correlates with cardiovascular and cerebrovascular diseases. Since the patients with PEX have a higher incidence of cardiovascular or cerebrovascular diseases, increased RDW can be detected in patients with an advanced form of XFS and with glaucoma [26]. Elevated inflammatory cytokines suppress the maturation of erythrocytes, and immature erythrocytes are detected in peripheral blood with increased RDW levels [27]. Some studies suggested that increased RDW levels correlate with the increased oxidative stress, which has been described in PEX patients. It is well known that oxidative stress additionally shortens the survival rate of erythrocytes.

Many inflammatory indices were established to predict the inflammatory processes in the body based on routine blood count results. If an ophthalmologist records an increase in monocyte and platelet counts, and a decreased lymphocyte count, they can predict that inflammation is included and that it will progress. All the indices for chronic inflammation were disturbed in our study. This can be very simply explained by using the data from earlier studies about the level of proinflammatory and regulatory cytokines in the process of PEX production [13,21]. The NLR ratio indicates that the elevated ratio in the process of PEX production is the result of an increased neutrophil count or a decreased lymphocyte count. Our results and the results of other studies reported a slight decrease in lymphocyte count, and an approximately similar neutrophil count. Neutrophil status can be explained by the fact that neutrophils are involved in acute inflammation, and that a decreased lymphocyte count is the result of being wasted with monocytes, the count of which is increased. We established a negative correlation between the lymphocyte and monocyte count in patients with PEX. Platelets regulate monocyte differentiation and activation [28]. They bind with monocytes and activate the inflammatory process. Earlier results indicated that the first step of chronic inflammation is in the early phase of XFS, and then chronic inflammation cytokines are increased in late stage XFS. The PLR index was also described as a very important index for some diseases, such as pancreatic adenocarcinoma and renal cell carcinoma [29]. We found statistically significant differences between these ratios in patients with late stage XFS and with XFG in comparison with other examined groups. These results indicated that platelets are very important cells in the process of chronic inflammation, as they activate the slow process of healing and activate the fibrotic process. The LMR index is used as a balance between an immune response and as a clinically relevant parameter in some malignant and inflammatory disease [18,19]. In our study, the LMR ratio was increased in patients with late stage XFS and XFG. We did not find any differences in the lymphocyte count, but the monocyte count was very important. Monocytes are the main cells in chronic inflammation, which is the basic process for the development of XFG. Our result did not indicate any differences in the lymphocyte count, so monocytes were the main cells in this kind of inflammation. There were no statistically significant differences in the neutrophil count, or in the lymphocyte count. Therefore, the inflammatory NLR index did not show any statistical significance. This is similar to earlier results [30]. Pathophysiologically, it can be explained as the part of chronic inflammation, which is the basic process in PEX production and XFG development. This inflammatory index was developed ten or more years ago, so certain other, more sensitive inflammatory indices are used for the prediction of patients with PEX material.

A great number of different studies suggested that a disturbed oxidative–antioxidative balance is the first step in patients with PEX [31,32]. This disturbed balance correlates with disturbed lipid peroxidation. Low HDL, elevated TG, elevated LDL, and elevated total cholesterol are described in patients with vascular damage. These results are controversial in patients with PEX. Janicijevic et al. indicated increased levels of cholesterol, TG, HDL and LDL in patients with XFS and XFG, without dividing XFS into early and late stage [11]. Kurtul et al. demonstrated that an increased level of LDL is statistically significant only in correlation with PEX patients [12], but Speckauskas et al. did not find a disturbed lipid profile [33]. In any case, a disturbed lipid profile is obviously to be expected in patients with PEX material, as they are at higher risk for cardiovascular and cerebrovascular diseases, especially patients with XFG. This group of patients is older, and they have other metabolic or vascular diseases, so the incidence of cardiovascular and cerebrovascular disease is higher.

Some other studies indicated similar results, as in the study of Dikmen and Un [34]. Their study included only 100 patients, divided into three groups, but we included 200 patients per group. We had four groups, as we divided the XFS group into early and late stages. Sarenac Vulovic et al. performed some earlier studies about cytokine levels in the process of PEX production and XFG [7,21]. This study is an extension of their earlier studies.

Our study examined, for the first time, these inflammatory indexes in patients with different stages of XFS, and included 200 patients per group. The limitation of this study is that it included only Caucasian patients.

This manuscript gives a lot of very useful information about patients with PEX (XFS/XFG) according to blood count test. A simple notification of monocyte, platelet and lymphocyte count while calculating immune indices can predict the course of the disease in consultation with different medical doctors, such as cardiologists, neurologists, vascular surgeons, etc.

## 5. Conclusions

Our results indicated statistically significant differences in monocyte and platelet count in all PEX groups in comparison with the control group, especially in late stage XFS and in XFG. Monocytes are the main source of various cytokines, including proinflammatory cytokines, so this result supports the proinflammatory theory of the development of pseudoexfoliation syndrome/glaucoma. Platelets play an important role in the differentiation and activation of monocytes, as well as in the process of chronic inflammation and fibrosis. There are already studies that indicate that PEX material is a product of chronic inflammatory response and the fibrotic process.

We also found that patients with late stage XFS had increased RDW values in comparison with patients with early stage XFS and the control group. Since RDW correlates with the development of cardiovascular and cerebrovascular disease, this finding indicates that patients with late stage XFS have a greater risk of developing these diseases.

An elevated NLR ratio in the process of PEX production is the result of an increased neutrophil count or a decreased lymphocyte count, because neutrophils are an important part of acute inflammation, while lymphocytes wasting into monocytes.

The LMR ratio was increased in patients with late stage XFS and XFG. We did not find any differences in the lymphocyte count. Our result shows that monocytes are the main cells in chronic inflammation, which is the foundation for the evolution of XFG.

The lipid profile analysis indicated increased values of LDL only in PEX patients. A disturbed lipid profile in these patients is expected, as they are at a higher risk for cardiovascular and cerebrovascular diseases.

## Figures and Tables

**Table 1:** Mean count of different blood cells from the hemogram.

Parameters	Early	Late	XFG	Control	*p*
**Leu**	7.1 ± 1.68	7.1 ± 1.59	7 ± 1.81	7.2 ± 1.74	*p* > 0.05
**Neu**	4 ± 0.78	4.1 ± 0.79	4 ± 0.81	3.9 ± 0.80	*p* > 0.05
**Mon**	0.76 ± 0.24	1.09 ± 0.31	1.11 ± 0.28	0.45 ± 0.21	*p* < 0.05
**Lymph**	2.1 ± 0.51	1.86 ± 0.57	1.85 ± 0.49	2 ± 0.47	*p* < 0.05
**MPV**	9.4	9.3	9.4	9.5	*p* > 0.05
**RDW**	13.9	14.7	14.8	14.1	*p* < 0.05
**Platelet**	251 ± 87	289 ± 86	301 ± 91	252 ± 81	*p* < 0.05
**Hol**	155.32	155.76	154.68	141	*p* > 0.05
**TG**	103.43 ± 21.23	105.54 ± 18.25	104.54	107.93	*p* > 0.05
**HDL**	44.65 ± 13.1	43.71 ± 12.24	45.62 ± 13.45	46.32 ± 15.81	*p* > 0.05
**LDL**	125.61	135.19	133.24	105.54	*p* < 0.05
**NLR**	1.9 ± 0.43	2.2 ± 0.41	2.16 ± 0.51	1.95 ± 0.46	*p* > 0.05
**PLR**	119.52 ± 34.2	155.38 ± 32.2	162.7 ± 39.2	126 ± 38.1	*p* < 0.05
**SIRI**	1.45 ± 0.42	2.4 ± 0.35	2.4 ± 0.37	0.88 ± 0.38	*p* < 0.05
**SII**	478.1 ± 185.6	637.04 ± 189.28	650.81 ± 191.25	491.4 ± 179.23	*p* < 0.05, *p* < 0.01
**SIMI**	90.84 ± 25.43	169.36 ± 31.12	180.6 ± 30.21	56.7 ± 26.65	*p* < 0.05, *p* < 0.01
**PIV**	363.35 ± 119.2	694.38 ± 124.25	722.4 ± 131.24	221.13 ± 109.12	*p* < 0.05, *p* < 0.01
**Mon/HDL**	0.017 ± 0.001	0.025 ± 0.0012	0.024 ± 0.0014	0.01 ± 0.009	*p* > 0.05
**Lymph/mon**	2.76 ± 0.12	1.71 ± 0.18	1.67 ± 0.23	4.44 ± 1.12	*p* < 0.05, *p* < 0.01

## Data Availability

Data is unavailable due to privacy.

## Abbreviations

The following abbreviations are used in this manuscript:

XFS	pseudoexfoliation syndrome
PEX	pseudoexfoliation
XFG	pseudoexfoliation glaucoma
NLR	neutrophil-to-lymphocyte ratio
PLR	platelet-to-lymphocyte ratio
LMR	lymphocyte-to-monocyte ratio
SII	systemic inflammatory index
SIRI	systemic inflammation response
IOP	intraocular pressure
CBC	complete blood count
RDW	red cell distribution width
CRP	C reactive protein
